# Structural characterization of the homotropic cooperative binding of azamulin to human cytochrome P450 3A5

**DOI:** 10.1016/j.jbc.2022.101909

**Published:** 2022-04-06

**Authors:** Mei-Hui Hsu, Eric F. Johnson

**Affiliations:** Department of Molecular Medicine, Scripps Research, La Jolla, California, USA

**Keywords:** cytochrome P450, cooperativity, drug metabolism, inhibition mechanism, protein structure, structure–function, X-ray crystallography, CYP3A5, CYP3A4, DMSO, dimethyl sulfoxide, PDB, Protein Data Bank, RSR, relative spectral response, SSRL, Stanford Synchrotron Radiation Lightsource

## Abstract

Cytochrome P450 3A4 and 3A5 catalyze the metabolic clearance of a large portion of therapeutic drugs. Azamulin is used as a selective inhibitor for 3A4 and 3A5 to define their roles in metabolism of new chemical entities during drug development. In contrast to 3A4, 3A5 exhibits homotropic cooperativity for the sequential binding of two azamulin molecules at concentrations used for inhibition. To define the underlying sites and mechanisms for cooperativity, an X-ray crystal structure of 3A5 was determined with two azamulin molecules in the active site that are stacked in an antiparallel orientation. One azamulin resides proximal to the heme in a pose similar to the 3A4–azamulin complex. Comparison to the 3A5 apo structure indicates that the distal azamulin in 3A5 ternary complex causes a significant induced fit that excludes water from the hydrophobic surfaces of binding cavity and the distal azamulin, which is augmented by the stacking interaction with the proximal azamulin. Homotropic cooperativity was not observed for the binding of related pleuromutilin antibiotics, tiamulin, retapamulin, and lefamulin, to 3A5, which are larger and unlikely to bind in the distal site in a stacked orientation. Formation of the 3A5 complex with two azamulin molecules may prevent time-dependent inhibition that is seen for 3A4 by restricting alternate product formation and/or access of reactive intermediates to vulnerable protein sites. These results also contribute to a better understanding of sites for cooperative binding and the differential structural plasticity of 3A5 and 3A4 that contribute to differential substrate and inhibitor binding.

Cytochromes P450 3A4, 3A5, and 3A7 contribute extensively to metabolic clearance of drugs (1, 2). P450 3A4 is expressed in adults, whereas 3A7 is generally expressed only in the fetal and perinatal period. Adult expression of 3A7 is seen, however, for carriers of *CYP3A7∗1C* allelic variant (3). Expression of the closely related 3A5 enzyme is expressed polymorphically in neonates and adults. In addition, 3A5 exhibits a wider tissue distribution, but its expression varies between ∼5% and ∼50% among biogeographic groups because of the prevalence of the *CYP3A5∗3* allele that leads to a loss of protein expression (4, 5). Individuals expressing a functional 3A5 may exhibit accelerated drug clearance for 3A4 substrates that reduces efficacy relative to population norms (6, 7, 8, 9).

Inhibition of the P450 3A enzymes can lead to problems for drugs that have narrow therapeutic windows. Understanding substrate and inhibitor interactions with P450s can provide information for drug redesign to avoid drug–drug interactions during drug development. X-ray crystal structures can define interactions of substrates and inhibitors with P450s at the atomic level as well as providing models for *in silico* studies of ligand binding. X-ray crystal structures have also defined the conformational plasticity of 3A4, which underlies its capacity to accommodate very large substrates as well as relatively small compounds.

Our laboratory published crystal structures indicating that the plasticity of 3A5 differs from that of 3A4. Ritonavir, an HIV protease and 3A4/3A5 inhibitor, adopts alternative conformations when bound in 3A5 (Protein Data Bank [PDB] code: 5VEU)^1^ compared with 3A4 (PDB code: 3NXU). Ritonavir causes the cavity to widen in 3A4 relative to the substrate/inhibitor-free structure when viewed with the heme as the base of the cavity. In 3A5, ritonavir binds in a narrower cavity with the distal end of ritonavir engaging the larger upper portion of the cavity (10). To better understand these conformational differences, the structure of 3A5 was determined in the absence of a substrate (PDB code: 6MJM). The 3A5 substrate-free structure exhibits a larger and more open cavity than that of substrate-free 3A4. Comparison of substrate-free structure of 3A5 to the ritonavir complex indicates that binding of ritonavir is coupled with conformational changes of 3A5 that close the cavity and maximize molecular interactions between the protein and substrate (11). In contrast, the active-site cavity of substrate-free 3A4 expands to accommodate ritonavir (12).

To expand our understanding of the differential structural plasticity between 3A4 and 3A5, we cocrystalized 3A5 with azamulin (MWY; ligand of interest), a semisynthetic antibiotic that is derived from the diterpene macrocycle of pleuromutilin by replacing a hydroxyl acetate group with an acetate connected to an amino-triazole ring *via* a thioether linker.^2^ In addition, the 6-ethenyl moiety of pleuromutilin was changed to an ethyl group (13). Azamulin is a relatively selective P450 3A inhibitor (14) that is used for reaction phenotyping. The structure of the 3A4–azamulin complex (PDB code: 6OOA) was determined with a single azamulin molecule bound in the active site (15). The pleuromutilin macrolide is positioned close to the heme, which leads to a type 1 spectral change in the visible spectrum for the Soret band because of reduction of water coordination to the heme iron (15). The type 1 shift lowers the wavelength of the Soret band, and the concentration dependence was characterized by a hyperbolic response with reported dissociation constants of 2 and 3 μM (14, 15).

In this study, cocrystallization of 3A5 with azamulin revealed that two molecules of azamulin are bound in the active-site cavity. In addition, a sigmoidal dependence of type 1 spectral shift is consistent with the binding of two azamulin molecules at concentrations used for inhibition studies. The proximal azamulin in the ternary complex with 3A5 is positioned like that seen for the binary 3A4–azamulin complex (PDB code: 6OOA), but the conformation of the acetate substituent differs between the two proteins. The distal azamulin molecule is stacked against the proximal azamulin in an antiparallel orientation. This orientation places the second macrocycle in the upper portion of the cavity with the amino triazole group positioned between Arg-106 and Ser-119 at the base of the helix BC loop. 3A4 exhibits both reversible and irreversible time-dependent inhibition by azamulin (14) with a relatively high inactivation rate/*K*_*i*_ of 0.4 μM/min (16). Nevertheless, time-dependent inhibition of 3A5 by azamulin was reported to be either very slow or nonexistent (17). The cooperative binding seen for azamulin with 3A5 may prevent mechanism-based inhibition by restricting sites of substrate metabolism and/or reduction of product reactions that lead to inactivation. In the absence of second azamulin molecule, the active site of 3A4 is much less restrictive and may allow alternative binding poses and/or expose additional vulnerabilities for adduction by a reactive azamulin metabolite. The underlying mechanism for time-dependent irreversible inhibition of 3A4 remains unclear. In addition to revealing differences in plasticity between 3A5 and 3A4, this structure reveals the basis for the homotropic cooperativity in the formation of the ternary complex of 3A5 with azamulin.

## Results

### Differences in azamulin binding between the four 3A5 protein chains in an asymmetric unit

**Figure 1:**
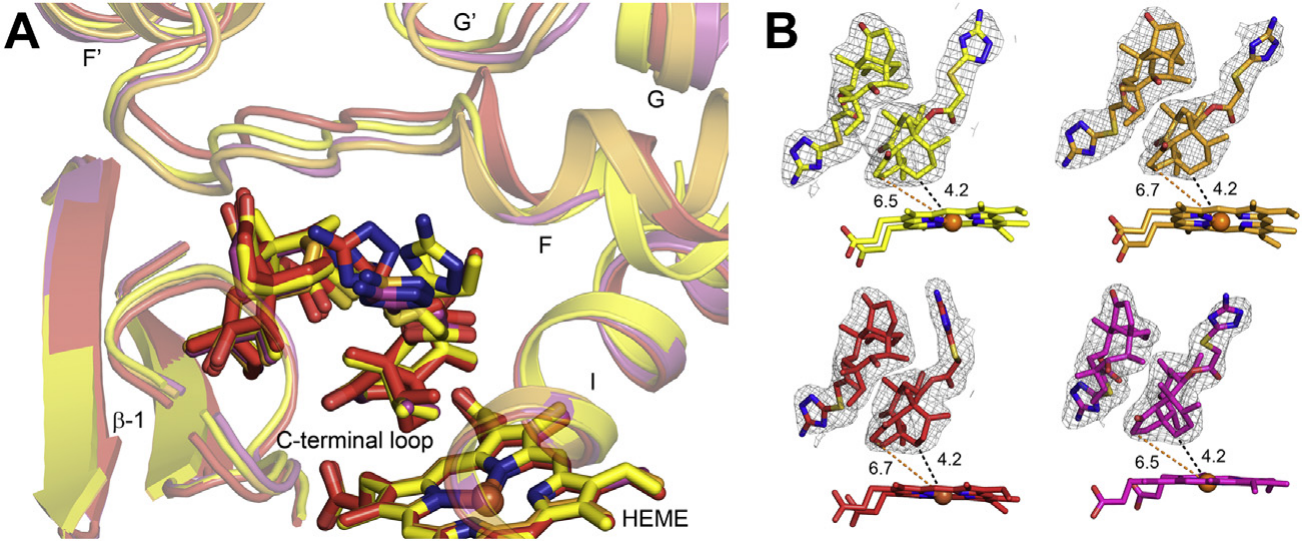
**Chains B (*light orange carbons*), C (*brick red carbons*), and D (*magenta carbons*) of the 3A5 ternary complex with azamulin superimposed on chain A (*yellow carbons*) using the align command in PyMol** (20) **with default settings.** Nitrogen, oxygen, sulfur, and iron atoms are colored *blue*, *red*, *mustard yellow*, and *orange*. *A*, displays differences in the conformations of the proximal and distal azamulin molecules and adjacent secondary structures. *B*, displays 2m*F*_o_–D*F*_c_ omit electron density maps for the azamulin molecules in each of the four chains. The distances (Å) between azamulin C9 and C15 and the heme iron are shown as *black* and *orange dashed line*, respectively. C9 and C15 correspond to major sites of hydroxylation for the related pleuromutilin antibiotics, tiamulin, valnemulin, and retapamulin, by 3A4 (13).

The 2.46 Å X-ray crystal structure of 3A5 complexed with azamulin in the C2221 space group revealed the presence of two molecules azamulin bound in each of the four chains of the asymmetric unit. Overlays of chains B, C, and D on chain A indicated that conformational differences for the distal azamulin molecule are unremarkable, but small differences were seen for the amino triazole moiety of the proximal azamulin (Fig. 1*A*). These confirmational differences and the locations of the molecules are well defined by unbiased 2m*F*_c_–D*F*_o_ omit maps (Fig. 1*B*). The amino triazole groups for the proximal azamulin extend out of the active-site cavity under the connecter between the F and F′ helices and reside between helix I and the C-terminal loop in an open channel that is designated the S-channel (18). These differences are likely to reflect structural differences between the four chains for the flexible connector between the F and F′ helices (Fig. 1*A*) that in turn reflect differences in lattice contacts for the different chains. The macrocycle of the proximal azamulin is positioned close to the heme surface with C9 centered 4.2 Å from the center of the heme iron (Fig. 1*B*). This distance is similar to the value of 4.27 Å seen in the structure of the 3A4–azamulin complex (PDB code: 6OOA). This close approach underlies type 1 shift seen for the binding azamulin in both enzymes. C9 and C15 of azamulin are prominent sites for hydroxylation of the related pleuromutilin antibiotics, tiamulin, valnemulin, and retapamulin, by 3A4 (13).

### Active-site interactions between 3A5 and azamulin

**Figure 2:**
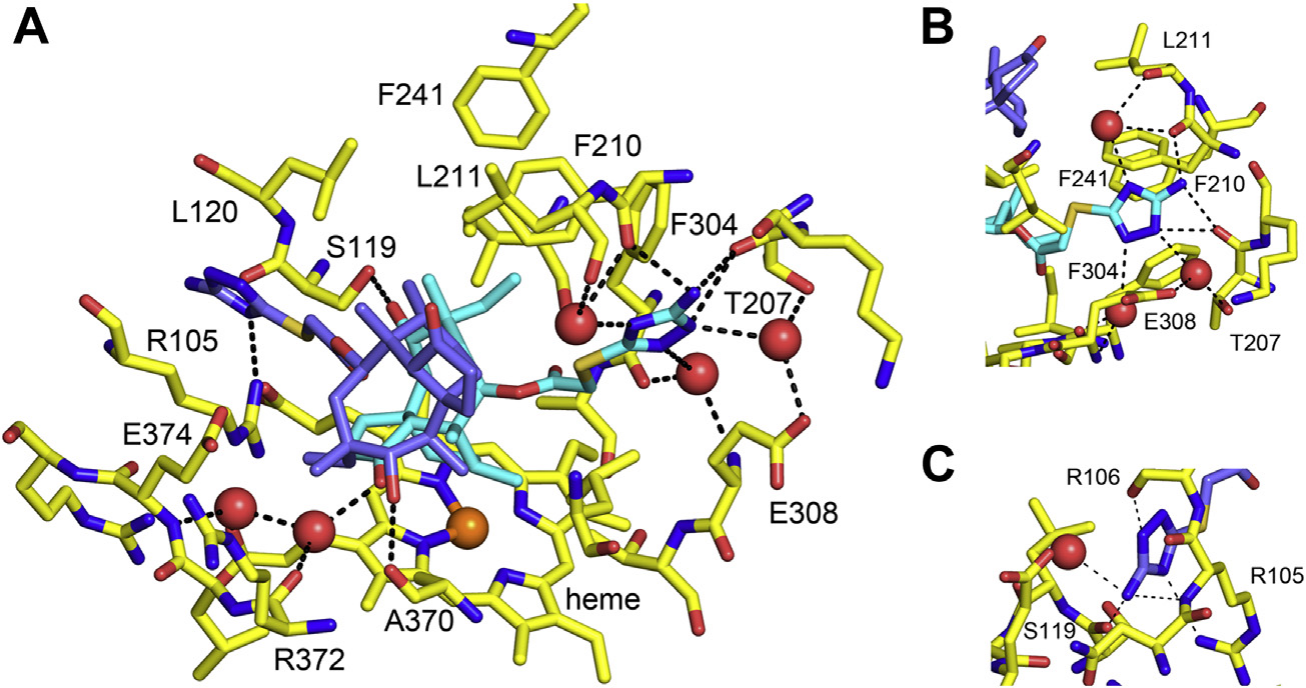
**Interactions of the proximal azamulin (*cyan carbons*) and the distal azamulin (*slate carbons*) and the heme are shown as *stick figures*.***A*, amino-acid residues that are within ≤4.5 Å of proximal azamulin in chain A are shown as *sticks figures*. In addition, water molecules that are hydrogen bonded to the azamulin molecules are shown as oxygen atoms as *red spheres* together with side chains that are hydrogen bonded to the water molecules. Hydrogen bonds (≥2.6 and ≤3.5 Å) are shown as *black dashed lines*. *B*, an alternative view of the hydrogen bonding to proximal azamulin amino triazole. *C*, a view of hydrogen-bonding interactions with the amino triazole moiety of the distal azamulin.

Chain A exhibits the lowest *B*-factors of the four chains and provides the most details regarding the role of solvation in the binding of azamulin. Interactions of the azamulin molecules with protein residues within ≤4.5 Å of the proximal azamulin in chain A are shown in Figure 2 with hydrogen-bonding interactions (*black dashed lines* ≥2.6 Å and ≤3.5 Å) between the protein and azamulin molecules or with bridging water molecules (oxygen shown as *red spheres*). Additional protein residues that are hydrogen bonded to the water molecules are also shown. The hydroxyl of the proximal azamulin donates an H-bond to Ser-119, and the amino nitrogen donates H-bonds to the carbonyls of Phe-210 and Thr-207. In addition, the triazole nitrogens of the proximal azamulin interact as donors or acceptors of H-bonds with water molecules (Fig. 2*B*), and the ketone group of the proximal macrocycle accepts an H-bond from a protein-sequestered water molecule near the heme surface (Fig. 2*A*). Some of the interacting water molecules are not evident or are reduced in number for other chains because of higher B values, differences in the surrounding residues, and orientations of the amino triazole group of proximal azamulin (Fig. 1*B*). The amino group on the triazole is unlikely to be charged at neutral pH because of conjugation with the triazole ring, which would impart sp2 character to the nitrogen and could also affect the hydrogen-bonding patterns. The hydroxyl group of the distal azamulin donates an H-bond to the carbonyl of Ala-370 (Fig. 2*A*). As shown in Figure 2*C*, Arg-105 donates an H-bond to N30 of the triazole of the distal azamulin, and N28 of the triazole donates an H-bond to the carbonyl of Arg-106. The amino group resides between the amide nitrogen of Arg-106 and the carbonyl of Ser-119 and could act as an H-bond donor and acceptor, respectively. The amino group can donate an H-bond to a water molecule seen in chain A, but this water molecule is not well defined by density in the other three chains.

Hydrophobic interactions contribute extensively to the stability of the complex by exclusion of water from the hydrophobic surfaces of azamulin and the protein. The buried surface area interactions between the azamulin, the protein, and heme were computed by PISA (Proteins, Interfaces, Structures and Assemblies) (19) using the PDBePISA server (https://www.ebi.ac.uk/pdbe/pisa/) (version 1.52). For the proximal azamulin in chain A, the buried surface area with the protein, the distal azamulin, and the heme are 422, 118, and 99 Å^2^, respectively, with an estimated ΔG of −10.2 kcal/mol. The estimated ΔG was the same for the distal azamulin with buried surface of 521 and 121 Å^2^ for the interfaces with the protein and proximal azamulin. These values are similar to that obtained for the single azamulin bound to 3A4 (PDB code: 6OOA), which yields estimated ΔG of −10.5 kcal/mol with buried surface of 452 and 111 Å^2^ with the protein and heme, respectively. As a result, the buried hydrophobic surface area for the 3A5 ternary complex is more than twice that of 3A4 binary complex with a significant contribution from stacking interactions of the proximal and distal azamulin molecules in 3A5, which likely contribute to the positive cooperativity.

### Structural comparison between 3A4 and 3A5

**Figure 3:**
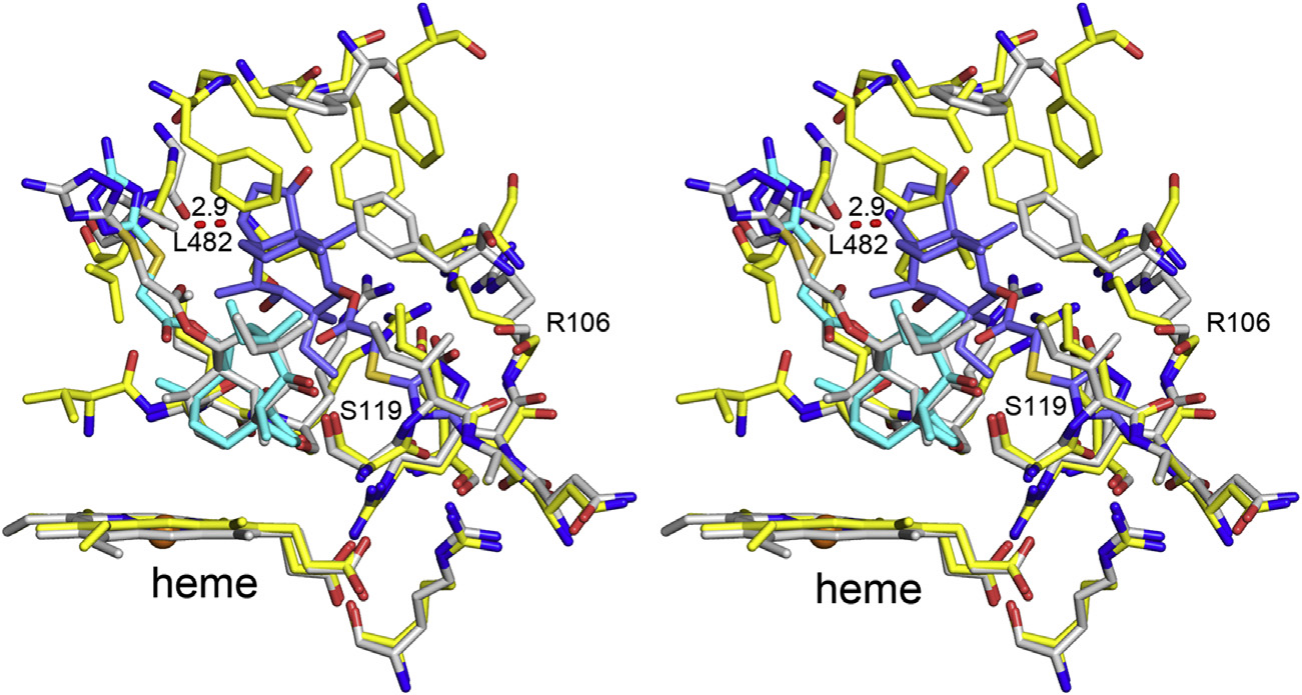
**The structure of the 3A4 binary complex with azamulin (PDB code:****6OOA****,*light gray carbons*) is superimposed on 3A5 chain A of the ternary complex (*yellow carbons*) and depicted in two panels for crosseye stereo viewing.** The 3A5 proximal and distal azamulin carbons are colored *cyan* and *slate*, respectively. Amino-acid residues that are within ≤4.5 Å of the 3A5 distal azamulin are displayed as *sticks* for 3A4 and 3A5. A clash (2.9 Å) between 3A4 L482 and C15 of the 3A5 distal azamulin is shown as *red dashed line*. Other 3A4 amino-acid residues overlap with 3A5 residues in lower portion of the cavity but reside outside the cutoff criterion for the upper cavity and form a larger solvent-occupied cavity in the 3A4 binary complex with azamulin. PDB, Protein Data Bank.

The structure of the 3A4 azamulin complex (PDB code: 6OOA) is superimposed on the 3A5 ternary complex in Figure 3 using the align command with defaults in PyMol (20). The binding of the proximal azamulin in 3A5 is very similar to the conformation seen for 3A4. 3A4 and 3A5 amino-acid residues residing ≤4.5 Å of the azamulin in distal cavity in the 3A5 ternary complex are shown in Figure 3 as *sticks*. The distal cavity in 3A4 is larger than that occupied by the distal azamulin in the 3A5 ternary complex, and in general, the selected 3A4 amino acids reside at similar distances from the 3A5 distal azamulin as those in the 3A5 complex or outside the 3A5 residues. The potential for binding a second molecule of azamulin in 3A4 would seem likely, but an exception is 3A4 Leu 482 CD2, which is 2.9 Å from C15 (*dashed red line*) of the distal 3A5 azamulin, which may disfavor the stacking interaction with the proximal azamulin in 3A4. In addition, the disordered residues in the helix F–F′ connector of the 3A4 azamulin complex (PDB code: 6OOA) could interfere with the binding of a second azamulin in 3A4 even though the F–F′ connector varies greatly between 3A4 structures. Nevertheless, the absence of a second azamulin suggests that movement of the azamulin is less restricted in the 3A4 active-site cavity and that solvent exposure of the azamulin in 3A4 binary complex is greatly increased relative to the 3A5 ternary complex.

### Structural plasticity of 3A5

**Figure 4:**
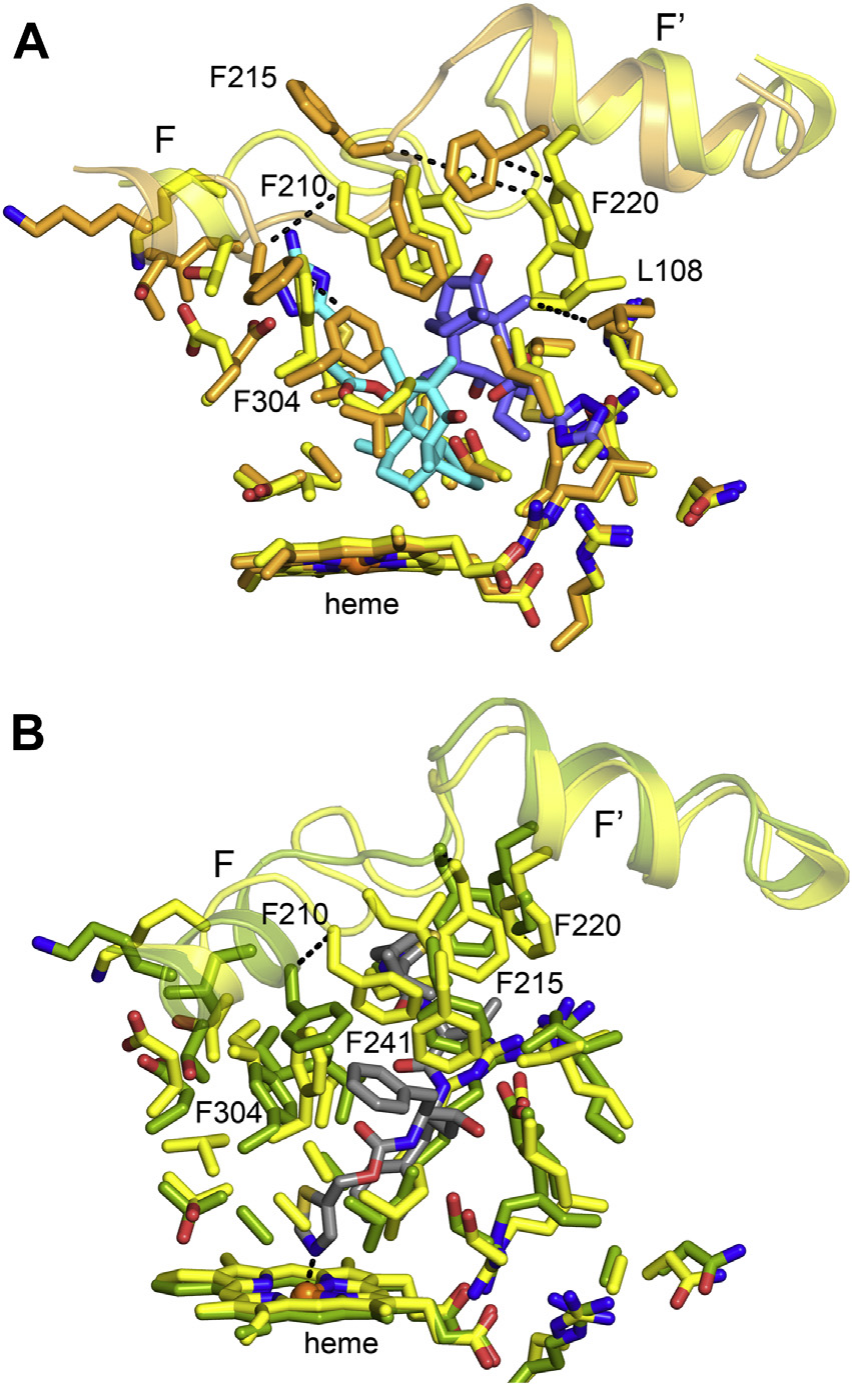
**P450 plasticity.***A*, superposition of the apo 3A5 (PDB code: 6MJM) structure (*orange carbons*) on chain A of the 3A5 ternary complex with azamulin (*yellow carbons*). Large changes are seen for L108, F304, F210, F215, and F220 among other changes. *B*, superposition of chain A (*green carbons*) of the 3A5 ritonavir binary complex on chain A of the 3A5 ternary complex. The positions of F304, F215, and F220 among other changes are like that for 3A5 ternary complex. Ritonavir (*gray carbons*) occupies the proximal and distal cavity, and the differences seen reflect differential induced fits for ritonavir and azamulin. PDB, Protein Data Bank.

Plasticity is gaged by the differences seen between the structure of the azamulin complex and the structure of the enzyme obtained in the absence of the ligand. The structure of the 3A5 apo protein (PDB code: 6MJM) exhibits a large cavity above the heme surface (11). A comparison to the conformation of chain A of azamulin complex indicates that the upper portion of the apo 3A5 active-site cavity reorganizes to engage the distal azamulin molecule (Fig. 4*A*). The proximal azamulin causes the F304 on helix I to rotate upward and displace the C-terminal end of helix F and the connector to helix F′, leading to displacement of F210 Cα by 4.8 Å. In addition, engagement of F215 with the distal azamulin reflects a 180° rotation of the side chain and a 6.8 Å translation of Cα. Large rotations are also seen for other side chains that exhibit smaller translations to interact with the distal azamulin. As seen when chain A of the 3A5 ritonavir complex is overlayed on chain A of the 3A5 ternary complex of azamulin in Figure 4*B*, similar but distinct changes occur in the distal cavity when ritonavir binds. In addition, ritonavir causes significant changes in the proximal cavity relative to apo structure to accommodate both the binding of thiazole to the heme iron and the stacking of the ritonavir P1 phenyl group above the thiazole moiety. In contrast, amino-acid side chains in the lower cavity do not exhibit significant differences between the azamulin complex and the apo structure. These differences between the azamulin ternary complex and the apo protein are much larger than the differences seen between the four chains in the asymmetric unit of the azamulin ternary complex. The plasticity of the upper active-site cavity provides a means to accommodate a wide range of compounds and more than one molecule at a time in the case of azamulin.

### Equilibrium binding of azamulin to 3A5

**Figure 5:**
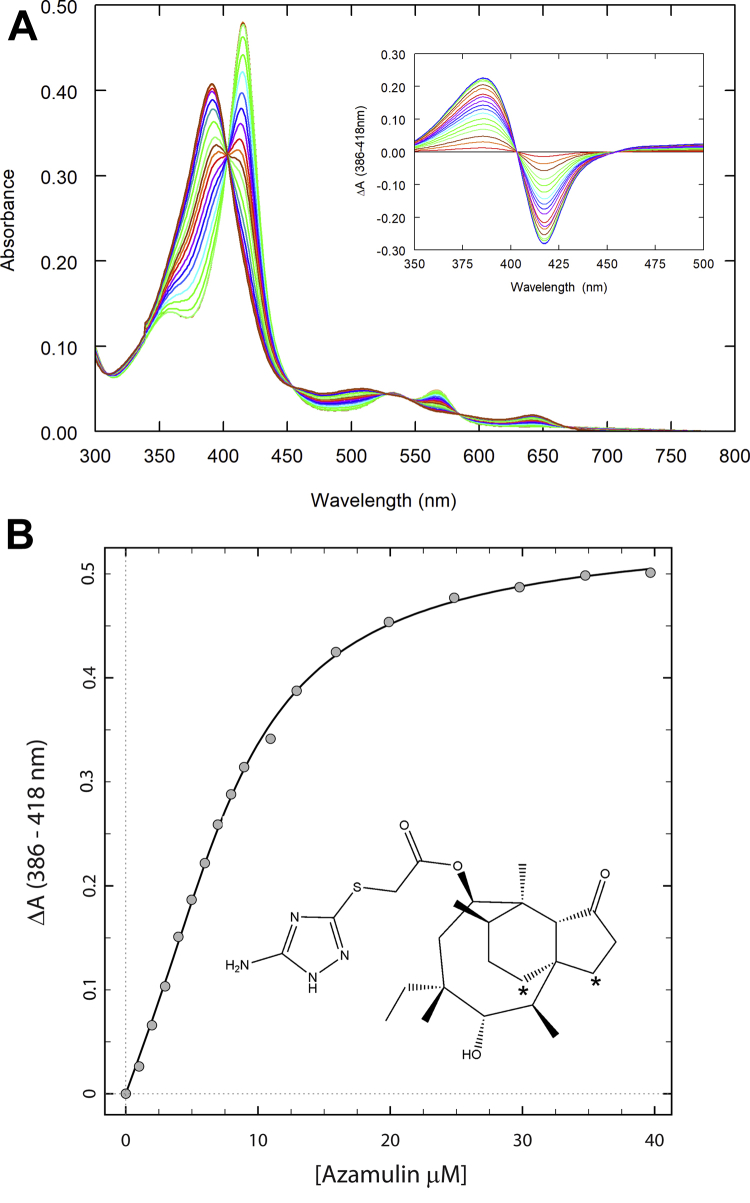
**Azamulin binding.***A*, titration of 3A5 (4.36 μM) with azamulin causes the Soret band at 418 nm to shift to 386 nm without significant residual low-spin character. Difference spectra shown in inset for the Soret band were computed by subtraction of the spectra from the spectrum recorded before addition of azamulin. *B*, the concentration dependence was fit numerically to a two-site sequential binding model. These results are representative of four replicate experiments. *K*_*d*_ and RSR values for the binary and ternary complexes are summarized in Table 1. A 2D structure of azamulin is depicted as an inset. The *asterisks* designate the closest carbons to heme iron. RSR, relative spectral response.

The binding of azamulin to 3A5 exhibits a blue shift of the Soret band in the UV–visible region of the P450 heme that is associated with an increase in high-spin character of the iron because of decreased occupancy of water to the sixth coordination site of the heme iron. This conversion is almost complete (Fig. 5*A*). This is consistent with the close approach of the proximal azamulin to the heme iron seen in the structure of the 3A5 ternary complex. The binding curve for 3A5 is sigmoidal, and a fit of the Hill equation to the data using nonlinear regression exhibited a *K*_*d*_ of 7.1 ± 0.4 μM with a Hill coefficient of 1.7 ± 0.1 (n = 4) using three different preparations of the protein at a nominal concentration of 4 μM. Under similar conditions, 3A4 exhibited a Hill coefficient of 1.0 and was well fit by the Morrison equation with a *K*_*d*_ of 2.0 μM (not shown), which is consistent with previous reports (14, 15). The sigmoidal changes for 3A5 indicate that higher concentrations of azamulin interfere with the binding of a water to the sixth coordination site of the heme iron because of the second azamulin molecule binding in the distal active site.

Dynafit (21) was used for additional fitting of the data to a sequential binding model. As the apparent *K*_*d*_ obtained from the fit of Hill equation is close to the concentrations of 3A5 used, the amount of protein-bound azamulin is likely to represent significant fraction of the total concentration of azamulin prior to saturation. In addition, the *K*_*d*_ determined for the Hill equation is an order of magnitude higher than the IC_50_ reported for inhibition of 3A5 enzymatic activity (14), suggesting that the first molecule to bind is likely to be inhibitory at a lower *K*_*d*_, but with a modest type 1 shift. The numerical approach compensates for depletion of free azamulin because of protein binding, which is not easily determined algebraically for models with two binding events. Figure 5*B* shows the fit for the sequential two-site binding model with the experiment data, and Table 1 summarizes results for four replicates. The relative spectral response 1 (RSR1) that relates the concentration of the binary complex to the mean change in absorbance is 0.031 ± 0.005, which is about a quarter of the value of RSR2 for the ternary complex, 0.12 ± 0.01. The apparent *K*_*d*_ for the initial binary complex in the sequential binding model is 0.65 ± 0.64 μM and 3.41 ± 0.60 μM for the ternary complex, which contributes most to spectral change. The *K*_*d*_ for the binary complex is similar to the IC_50_ reported for azamulin inhibition of 3A5 by Stresser *et al.* (14). The *K*_*d*_ values for the intermediate binary complex exhibit higher variation (Table 1) between independent experiments because of its low contribution to spectral change. The recommended concentration for *in vitro* characterization of azamulin inhibition is 5 μM (14), suggesting that the ternary complex would be present in normal usage for reaction phenotyping. This azamulin inhibition of 3A5 is considered to be reversible but may exhibit mixed mode if a small substrate binds without displacing both molecules of azamulin.

**Table 1:** Binding characteristics for pleuromutilin antibiotics with 3A5 using type 1 spectral shift

Ligand	Azamulin	Lefamulin	Pleuromutilin	Retapamulin	Tiamulin
[P450] (μM)	3.73–4.36	1.90–1.92	1.80–1.98	2.01–2.03	0.94–1.98
Equation/model^a^	4	2	2	2	3
*K*_*d*_ (μM)		55.1 ± 7.8	32.7 ± 4.3	16.6 ± 2.4	6.31 ± 1.02
Δ*A*_max_/[P450]		0.06 ± 0.00	0.12 ± 0.02	0.09 ± 0.00	0.08 ± 0.00
*K1*_*d*_ (μM)	0.65 ± 0.60				
RSR1^b^	0.03 ± 0.01				
*K2*_*d*_ (μM)	3.41 ± 0.60				
RSR2^b^	0.12 ± 0.01				
Replicates^c^	4	2	3	2	4

a Equations and models are described in the Experimental procedures section.

b RSR1 and RSR2 relate the concentrations of the binary and ternary complexes to the observed ΔA, respectively. When both binding sites are saturated, RSR2 can be compared with Δ*A*_max_/[P450] for one-site binding.

c Values of *K*_*d*_, RSR, and Δ*A*_max_/[P450] are means. The variance is a SD for three or four replicates, and for two replicates, the variance is the deviation of replicates from the mean.

### Equilibrium binding of tiamulin and other pleuromutilin antibiotics to 3A5

We examined whether the pleuromutilin antibiotic tiamulin would exhibit the homotropic cooperative binding. Tiamulin was a precursor of azamulin that was found to alter the metabolism of other drugs when used in livestock. Subsequent studies determined that tiamulin did so by inhibition of family 3A P450s (22, 23). The semisynthetic pleuromutilin antibiotics, tiamulin, valnemulin, and retapamulin, have larger substituents than the amino triazole group of azamulin with tiamulin being the closest in size to azamulin. They also have a 6-ethenyl group instead of the 6-ethyl moiety of azamulin. Human microsomal oxidative metabolism of these three related antibiotics is selective for P450 3A among the major hepatic drug–metabolizing P450s tested (13), and hydroxylation of the 8α and 2β C–H bonds lead to the predominant metabolites. Azamulin C9, which resides closest to the heme iron, corresponds to C8 of tiamulin, and azamulin C14 corresponds to tiamulin C2. Azamulin C14 resides 6.5 to 6.7 Å from the heme iron depending on the chain. In addition, lefamulin (Xenleta), which was recently approved for the treatment of community-acquired pneumonia, is contraindicated for patients taking drugs that are metabolized by 3A enzymes, which might cause long QT (24). The Food and Drug Administration submission indicates that lefamulin is also metabolized by 3A4 and 3A5 among the P450s tested, and the primary metabolite is 2R-hydroxy-lefamulin (25), as seen for tiamulin. Another hydroxylated metabolite was detected, but the site of the hydroxylation was not reported.

**Figure 6:**
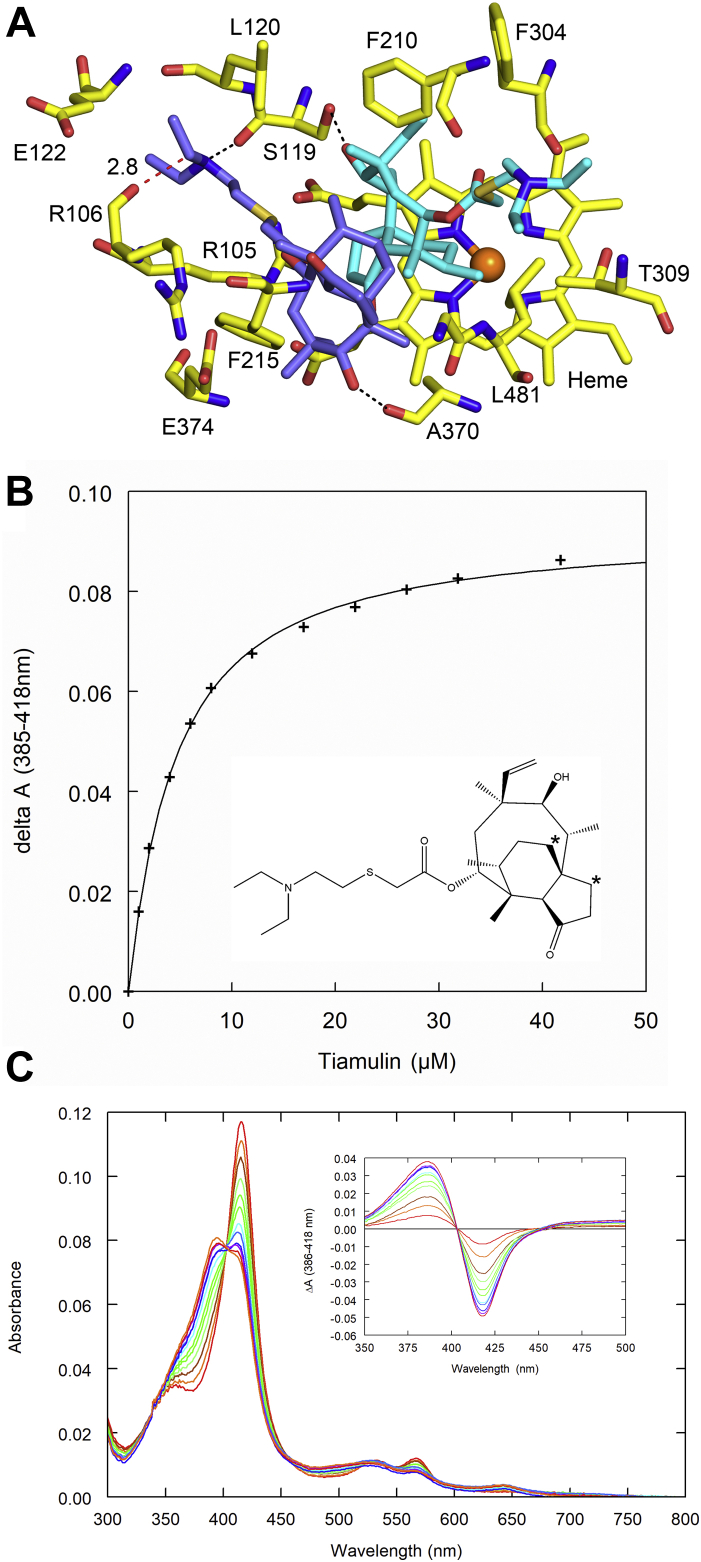
**Tiamulin binding.***A*, tiamulin modeled in chain A of 3A5 ternary complex with azamulin. A 2D structure of tiamulin is inset in (*B*), and the *asterisks* designate prominent sites of hydroxylation. Tiamulin differs from azamulin by substitution of ethane with for the ethylene on the azamulin macrocycle and substitution of amino triazole moiety at the terminus of thioether with a triethyl amino group. Coot was used to overlay tiamulin in the electron density for the proximal azamulin (*cyan carbons*) and the distal azamulin (*slate carbons*) followed by manual adjustments to reduce clashes with the triethyl amino moiety of tiamulin. Amino-acid residues ≤3.5 Å from the tiamulin molecules are shown as *stick figures*, as are tiamulin and heme. Hydrogen bonds are shown as *black dashed lines*, and an atomic clash ≤3.0 Å is shown by a *red dashed line*. The terminal group of the thioether is not accommodated well for distal tiamulin because of the larger size and loss of the planarity seen for azamulin. The protonated nitrogen of the distal tiamulin is positioned to donate an H-bond to the carbonyl of Ser-119, the carbonyl of Arg-106 is 2.8 Å from tiamulin C25, and the ethyl groups exhibit eclipsed conformations. In contrast, the larger triethyl amino group was accommodated for the proximal tiamulin as it extends into the S-channel cavity. *B*, in contrast to azamulin, the concentration dependence of type 1 spectral shift for tiamulin using 1.05 μM 3A5 exhibits a hyperbolic fit to a tight-binding one-site model. *C*, there is evidence for a significant residual low-spin component at saturation. *K*_*d*_ and Δ*A*_max_/[P450] values are summarized for four replicates in Table 1.

A manual overlay of tiamulin on the proximal azamulin in the ternary complex with 3A5 suggested that tiamulin could bind like the proximal azamulin with the triethylamine group extending further into the solvent channel formed by F–F′ connector, helix I, and the C-terminal loop. This also places the 8α and 2α C–H bonds close to the heme iron as seen for azamulin. On the other hand, when superimposed on the distal azamulin, the longer and less planar triethylamino moiety conflicts with amino-acid residues that form the slot that binds the planar aminotriazole group of azamulin (Fig. 6*A*). Consistent with superposition of tiamulin on the proximal azamulin, tiamulin exhibits type 1 spectral shift, but the concentration dependence for binding is hyperbolic (Fig. 6*B*) rather than sigmoidal with an estimated *K*_*d*_ of 6.31 ± 1.0 μM (Table 1), which is similar to an IC_50_ of 1.69 μM for inhibition of human microsomal testosterone turnover by tiamulin (26) and midazolam turnover to 1-hydroxy-midazolam (27). In contrast to azamulin, type 1 spectral shift obtained with tiamulin leads to a bifurcated Soret band indicating a residual low-spin content at saturation (Fig. 6*C*) and a lower Δ*A*_max_ normalized to 3A5 concentration. This is likely because of increased dynamics of pleuromutilin group or alternative orientations for tiamulin binding. The lack of evidence for homotropic cooperativity for tiamulin binding to 3A5 and the lower efficacy for type 1 spectral shift suggests that the distal azamulin enhances efficacy for type 1 spectral change by restricting the proximal azamulin to reside close to the heme iron.

**Figure 7:**
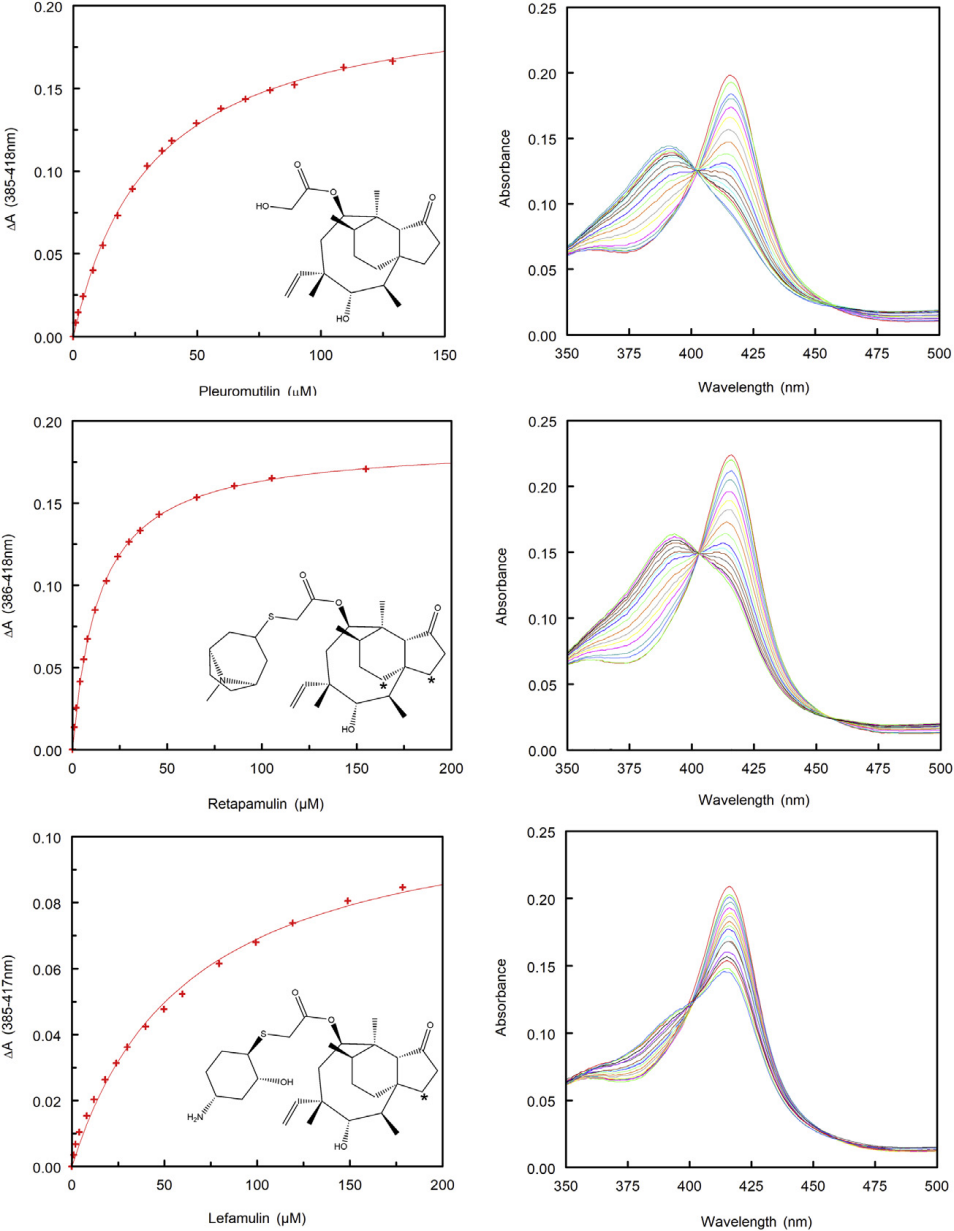
**Representative type 1 spectral binding isotherms (*left*) and spectral shifts for the Soret peak (*right*) are depicted for pleuromutilin, retapamulin, and lefamulin with 1.80, 2.03, and 1.90 μM P450 3A5, respectively.** 2D structures of the ligands are inset. *Asterisks* identify known prominent sites of hydroxylation for retapamulin and lefamulin. A one-site ligand-binding model was fit to the data, and the values of mean *K*_*d*_ and Δ*A*_max_/[P450] for two or three replicates are summarized in Table 1.

We also characterized the binding isotherms for pleuromutilin, the semisynthetic topical antibiotic retapamulin (Altrax), and the semisynthetic systemic antibiotic lefamulin (Xenleta). These three isotherms were also hyperbolic (Fig. 7) with mean *K*_*d*_ values of 32.7 ± 4.3, 16.6 ± 2.4, and 55.1 ± 7.7 μM, respectively (Table 1). Like azamulin, pleuromutilin, which lacks the thioether side chain, exhibited type 1 shift without significant residual low-spin component, whereas similar to tiamulin, retapamulin and lefamulin exhibit partial type 1 shifts with Soret peaks with well-defined shoulders indicating that their side chains affect the positioning of the pleuromutilin group near the heme (Fig. 7). A shoulder is also evident for type 1 shift for the binding of azamulin to 3A4 (15), which is not evident for the 3A5 ternary azamulin complex (Fig. 5*A*), suggesting that distal azamulin restricts the dynamics of the proximal azamulin.

## Discussion

Azamulin was used for cocrystallization with 3A5 to provide additional information regarding the differential plasticity between 3A5 and 3A4. Structures of the two substrate-free proteins exhibit very different substrate-binding cavities with 3A5 exhibiting a higher ceiling above the heme and narrower active site than 3A4. The lower ceiling for 3A4 reflects extensive interactions between aromatic side chains that close the ceiling and a propensity for Arg-212 on helix F to donate a hydrogen bond to the carbonyl Phe-304 on helix I in the active site. Azamulin was shown to disrupt some of these interactions when it occupies 3A4 active-site cavity and displaces the connector between helix F and F′ helices. These changes were localized and did not cause extensive conformation changes in the structure of 3A4 (15). We suspected that the different size and shape of the active-site cavity of substrate-free 3A5 might bind azamulin differently and/or exhibit larger conformational changes of the protein to optimize interactions with azamulin as seen when ritonavir is bound to 3A5 (11). The binding of one molecule of azamulin proximal to the heme surface of 3A5 positions pleuromutilin macrocycle like that seen for the structure of the 3A4 azamulin binary complex (PDB code: 6OOA) with some differences to accommodate the azamulin acetate side chain because of differences between the two proteins in the differential plasticity of the helix F–F′ region of each protein.

The accommodation of a second azamulin molecule in the 3A5 active-site cavity is the major difference from 3A4. Large conformational changes relative to substrate-free structure were evident to accommodate the ternary complex and increase hydrophobic interactions with the azamulin molecule in the upper regions of the cavity. This was also seen for the structure of the 3A5 ritonavir binary complex (PDB code: 5VEU), which occupies both the proximal and distal portions of the active site (11), with differences to enhance interactions with each compound in the distal cavity, suggesting significant induced fits for each compound.

When it binds to 3A5, azamulin elicits a sigmoidal concentration-dependent type I spectral shift with a mean Hill coefficient of 1.7 and *K*_*d*_ of 7.1 μM. As the protein concentration was close to the *K*_*d*_ value, depletion of azamulin by protein binding is likely to confound the interpretation of the results for the Hill equation. Further characterization using numerical methods with a sequential binding model that accounts for free substrate depletion because of tight binding led to estimated *K*_*d*_ values of 0.6 and 3.4 μM for the first and second binding equilibria with relative spectral responses of 0.03 and 0.12, respectively. The formation of the ternary complex led to a fourfold greater effect on type 1 shift than estimated for the binary complex. The magnitude of the type 1 shift is almost complete, which is consistent with the close approach of the proximal azamulin to the heme that is positioned similarly to that seen for the structure of the 3A4 azamulin complex. Both the proximal and distal azamulin-binding sites exhibit beneficial polar interactions with the azamulin and diminished solvation of hydrophobic surfaces of azamulin and 3A5. In addition, the antiparallel orientation of the long axis of the azamulin molecules in 3A5 provide a strong additional hydrophobic interaction that likely contributes to the positive cooperativity of type 1 shift. To our knowledge, this is the first 3A5 X-ray crystal structure revealing the basis for positive homocooperative binding.

Restrictions on the length of the long axis of the distal azamulin suggested that the increased length of the terminal moiety of the thioether group for tiamulin and lack of planarity would disrupt cooperative binding. Binding isotherms for tiamulin and 3A5 exhibit a hyperbolic concentration dependence with a similar *K*_*d*_ to azamulin. Although our modeling of tiamulin in the structure of ternary azamulin complex indicated that the pleuromutilin group of tiamulin could be accommodated as seen for the proximal azamulin, the maximum amplitude of type 1 shift indicated a reduced capacity to displace water from the sixth coordination site of the heme iron. The latter suggests that binding conformation might be less constrictive because of the loss of steric effects in the absence of the second molecule in the distal cavity. This was also observed for the topical antibiotic retapamulin (Altrax) and the systemic antibiotic lefamulin (Xenleta), which have longer and less planar thioether groups. In contrast, pleuromutilin, which lacks a thioether substituent, exhibits a larger type 1 shift but does not exhibit evidence for cooperative binding.

**Figure 8:**
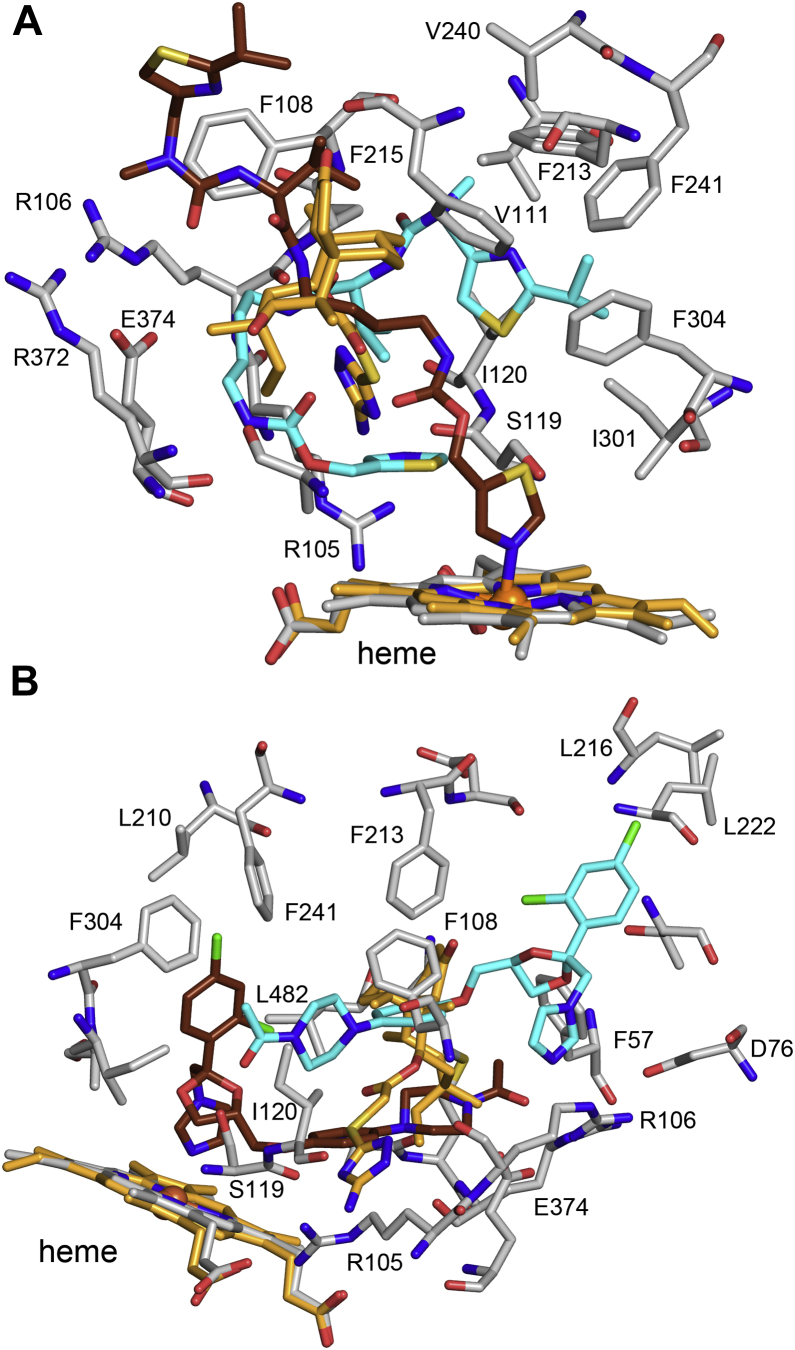
**Structures of 3A4 ternary complexes.***A*, an analog of ritonavir GS4 (1RT) and (*B*) ketoconazole (KLN) overlaid on chain A of the 3A5 ternary complex with azamulin (PDB code: 7SV2). 3A4 carbons are colored *gray*, and 3A5 heme and distal azamulin carbons are colored *orange*. Carbons for proximal and distal 3A4 inhibitors are colored *brown* and *cyan*, respectively. 3A4 residues ≤4 Å from the distal GS4 and both molecules of ketoconazole are shown as *stick figures*. The long axis of the distal azamulin in 3A5 runs perpendicular to the distal GS4 and both ketoconazole molecules in the 3A4 ternary complexes.

Multiple occupancy by substrates and inhibitors in the active site is often evoked to explain binding equilibria and kinetic data exhibiting both homocooperativity and heterocooperativity for P450 family 3A enzymes (28, 29, 30). In addition, an external allosteric site has been considered based initially on X-ray crystal structure of 3A4 (PDB code: 1W0F) with a molecule of progesterone (STR) bound in an external binding site above the ceiling of the active site near the outer surfaces of the G and G′ helices (31), but X-ray crystal structures for ternary complexes with molecules that exhibit cooperative binding are rare (30). A notable example from a study by Sevrioukova and Poulos (32) is a ternary complex of 3A4 with a ritonavir analog GS4 (1RD), which lacks the two phenyl groups of ritonavir leading to increased flexibility and a reduction in size. The X-ray crystal structure of the 3A4 ternary complex of GS4 (PDB code: 4K9T) revealed relatively localized changes from the conformation of substrate-free structure of 3A4. The localized differences include displacement of the Arg-212 from the cavity, adoption of an alternative rotamer for Phe-304 that is associated with raising the helix F–F′ connector as seen also for the binding of azamulin to 3A4 and 3A5. The thiazole nitrogen of the proximal GS4 is bound to the heme iron, and the proximal GS4 extends out to the surface in orthogonal direction relative to that of the long axis of the distal azamulin in 3A5 (Fig. 8*A*). This reflects in part the wider active-site cavity in 3A4 relative to 3A5 that remains open in the 3A4 azamulin binary complex. The thiazole of the distal GS4 stacks above the heme near S119 in the helix B′–C connector and loops over the proximal GS4 to place the distal end near F304 on the I helix in the proximal cavity. The binding of thiazole group to the heme causes type 2 spectral change that is biphasic and characterized by *K*_*d*_ values of 1.5 and 13.1 μM. The first binding event imparts the largest portion of type 2 shift with second binding event augmenting type 2 shift to a lesser degree (32). This suggests that binding of the second GS4 may alter the interaction of the proximal GS4 thioazole group with the heme iron.

An earlier X-ray crystal structure determined by Ekroos and Sjogren (33) for a ternary complex for 3A4 with two molecules of ketoconazole bound in the active site (PDB code: 2V0M) was pivotal for our early understanding of 3A4 plasticity and the potential for more than one molecule to bind in the 3A4 active site. The protein conformation of the complex showed large changes relative to the substrate-free structure of 3A4 (33), and the conformation of the (PDB code: 2V0M) structure more closely matches the conformation of 3A5 substrate-free structure (PDB code: 6MJM) (11). Ketoconazole is a tight binding inhibitor of 3A4 that produces type 2 spectral shift because of the coordination of its azole nitrogen to the heme iron. A study by Isin and Guengerich (34) used type 2 shift to characterize the binding of ketoconazole to 1 μM 3A5 and observed a biphasic hyperbolic response. Concentrations of ketoconazole below 1 μM exhibited tight binding with an apparent 1:1 stoichiometry and a second phase that plateaued at a 2:1 stoichiometry. The data were better fit by the Hill equation with Hill coefficient of 1.4 than a tight binding one-site model. These results suggest that the binary complex with ketoconazole predominates at 3A4 concentrations <1 μM, and the second phase suggests a potential transition to the ternary complex at concentrations of ketoconazole greater than 1 μM.

The two ketoconazole molecules are bound in antiparallel configuration like that seen for the two azamulin molecules bound to 3A5. The proximal ketoconazole is bound to the heme iron by the azole nitrogen and is accommodated in extra width of the 3A4 active site, which is orthogonal to the long axis of the distal azamulin in 3A5 (Fig. 8*B*). The second ketoconazole is stacked above the proximal ketoconazole in antiparallel orientation in an expanded distal active-site cavity with the dichlorophenyl moiety in the channel opening between the A′ and F′ helices. Molecular dynamics simulations and free-energy calculations indicated that shape complementation and van der Waals interaction between the two ketoconazole molecules were likely to contribute the predicted homotropic cooperativity (35). These observations are likely to apply to the 3A5 ternary complex with azamulin because of the large reduction in solvent exposure in the active-site cavity and for the ligands because of a similar and extensive stacking interaction, as discussed earlier.

The sequential binding model reflects the binding of first and second molecules of azamulin in the orthostatic cavity. The first binding event may reflect the binding of azamulin in the distal cavity followed by relocation to the proximal site. This was reported by Hackett (36) for an accelerated molecular dynamics simulations of the binding of testosterone to membrane-imbedded 3A4, where the testosterone resides initially in the distal cavity under the G′ helix with its long access orthogonal to the heme followed by a more stable binding near the heme surface with the long axis of testosterone parallel to the heme surface (36). On the other hand, azamulin is a much larger molecule, and first molecule might also bind near the heme followed by the binding of second azamulin in distal cavity, causing the proximal azamulin to move closer to the heme iron.

For the most prominent P450s in hepatic metabolic drug clearance, ketoconazole is a relatively selective inhibitor for *in vitro* reaction phenotyping, but azamulin is a more selective inhibitor of family 3A P450s when screening additional P450s (14). The IC_50_ concentrations for azamulin with 3A4 are ∼100-fold lower than the *K*_*d*_ estimated by type 1 spectral shifts, and these lower values are likely to reflect time-dependent irreversible inhibition of 3A4 rather than reversible binding (14, 16, 37). The underlying mechanism for the time-dependent inactivation of 3A4 by azamulin remains unknown. In contrast, time-dependent inhibition of 3A5 by azamulin was reported to be either very slow or nonexistent (17). Binding of the distal azamulin may prevent conversion of proximal azamulin to a reactive intermediate capable of inactivation of the enzyme. The IC_50_ values reported for competitive inhibition of recombinant 3A5 (14) are more similar to the *K*_*d*_ obtained for formation of the initial binary complex than for the ternary complex. Although the *K*_*d*_ for formation of the ternary complex is higher, it is similar to concentrations of azamulin that are generally used for inhibition studies. In addition, the potential for azamulin to bind in the distal site might lead to mixed modes of inhibition for some substrates that can coexist with a distal azamulin in the active site.

In summary, the binding of azamulin in the 3A5 active site differs from that seen for 3A4 in that it exhibits homotropic cooperativity. The distal azamulin binds in a large extension of the orthosteric active site that resides under helix G′ in the substrate-free structure, and when bound, the plasticity of 3A5 contributes to reorganization of amino-acid side chains to stabilize the ternary complex. In contrast, 3A4 binds one molecule of azamulin with smaller changes relative to the substrate-free structure and with a significant residual portion of the orthosteric cavity accessible to solvent. The large interaction surface between the distal and proximal azamulin molecules contributes to the stability of the ternary complex and may contribute to lower propensity for time-dependent inhibition of 3A5 to larger steric constraints on the proximal azamulin relative to 3A4.

## Experimental procedures

### Protein structure determination

P450 3A5 was expressed without its N-terminal transmembrane helix and short luminal extension (Δ3-24). In addition, five amino acids at the C terminus were replaced by a 4-histidine tag (10). This modified protein, 3A5C2dH, was expressed in *Escherichia coli* and purified as described previously (11). The reduced CO *versus* reduced P450 difference spectra were used to assess the concentration of 3A5 (38). The protein was concentrated to 1.2 mM in 50 mM sodium Hepes (pH 7.4), 50 mM potassium acetate (pH 7.4), 20% glycerol, 0.5 mM EDTA, and 10 mM β-mercaptoethanol. Formation of the azamulin (Cayman Chemical) complex was based on the protocol used to crystallize the 3A4 azamulin complex (15) by combining the highly concentrated protein with ∼3× molar ratio of azamulin from a 50 mM stock solution in dimethyl sulfoxide (DMSO). After a 75 to 105 s incubation, the sample was spun for 10 s in a microcentrifuge, and the supernatant was used for crystallization. Crystals of the azamulin complex were obtained by sitting-drop vapor diffusion at 23 °C. Drops contained 0.6 μl protein solution, 0.1 μl 18 mM *n*-decyl-β-d-maltoside (Anatrace), and 0.5 μl well solution containing 0.2 M sodium malonate (pH 6.5) and 30% PEG3350. A 10 s soak in Paratone-N was used for cryoprotection before placing the crystal in liquid nitrogen.

An initial 3.18 Å dataset exhibiting the C 2 2 21 space group was phased by molecular replacement using PHASER (39) and the 3A5 substrate-free structure (PDB code: 6MJM) as a probe. The solution exhibited a translation function Z-score of 27.4 and a log-likelihood gain of 667 with four chains in the asymmetric unit. An initial model was built for chain A and refined using noncrystallographic symmetry to build the four chains in the asymmetric unit. Subsequently, a 2.3 Å dataset was collected from a single crystal at the Stanford Synchrotron Radiation Lightsource beamline 12-1 at 100 K using Blu-Ice. The data were indexed and integrated using XDS (40) followed by merging and scaling using Aimless (41). The high-resolution cutoff for data refinement reflected a CC1/2 ≥10% and a mean *I*/SD of ∼1. The data were phased by isomorphic replacement using the earlier model obtained using the 3.18 Å dataset. The free R flags were maintained for the initial and final datasets using ≈5% of unique reflections. The limiting resolution for final model refinement was based on a paired refinement method (42) as implemented by the PDB_REDO webserver (43). This analysis recommended a high-resolution limit of 2.47 Å for model refinement. The PDB_REDO server also optimized translation-libration-screw-rotation parameters and improved aspects of the model. This model was further improved by adjustments using Coot software (44) and refinement using Phenix (45) to a resolution of 2.46 Å, which corresponds to a resolution bin reported by Aimless for the data refinement statistics. Chains A and B are the most complete with only one residue missing at the N terminus, six and five missing residues at the C terminus, and eight and nine missing residues in the connector between the H and I helices, respectively. The C and D chains also exhibit conformational disorder that includes the portions of the connecter between the G and H helices in addition to five residues at the N terminus. A portion of the connecter between the E and F helices in chain D could not be modeled. The more extensive confirmational disorder in chains C and D is likely to reflect sparse crystal contacts in these regions relative to that of chains A and B. The statistics for the data refinement and structure determination are provided in Table 2. Attempts to crystallize a 3A4 ternary complex using similar concentrations of azamulin and protein produced crystals of the binary complex when phased by molecular replacement with the structure of the 3A4 azamulin complex (PDB code: 6OOA).

**Table 2:** Data collection and model refinement statistics

Data collection		Model refinement	
Resolution range (Å)	39.56–2.30	Resolution range (Å)	34.274–2.46 (2.50–2.46)
	[2.51–2.46]^a^		
	(2.34–2.30)^b^		
Space group	C 2 2 21	Reflections	70,652 (2804)^b^
Unit cell lengths (Å) Angles °	104.79 134.30 275.27 90 90 90	R-free reflections	3400 (147)^b^
Wavelength (Å)	0.97946	R-work	0.2092 (0.2512)^b^
Total reflections	1,037,643 (7504)^b^	R-free	0.2480 (0.3074)^b^
Unique reflections	85,964 (4291)^b^	Heavy atoms	15,215
Multiplicity	12.1 [12.3]^a^ (9.7)^b^	Ramachandran favored (%)^c^	96.89
Completeness (%)	99.6 [100]^a^ (95.6)^b^	Ramachandran outliers (%)^c^	0.00
CC1/2	0.996 [0.599]^a^ (0.202)^b^	Average *B*-factor (Å^2^)	63.54
Mean (I/SD)	7.8 [1.6]^a^ (0.8)^b^	Protein (Å^2^)	64.18
Wilson *B*-factor (Å^2^)	44.80	Ligands (Å^2^)	46.43
		Waters (Å^2^)	48.42
		Translation-libration-screw-rotation groups	1/Chain

Cytochrome P450 3A5 (CYP3A5) azamulin ternary complex Worldwide PDB code: 7SV2.

b ( ) Values for the highest resolution shell for data and model refinement.

a [ ] Values for highest resolution data shell used for structure refinement.

c Ramachandran results were analyzed using MolProbity in Phenix.

### Spectral binding studies

Type 1 spectral changes elicited by increasing concentrations of a ligand were determined by recording spectra from 800 to 250 nm using a Cary 100 dual-beam UV–visible spectrophotometer. The sample cuvette contained 3A5, and the corresponding buffer was in the reference cuvette. The protein buffer consisted of 50 mM sodium Hepes at pH 7.4, 50 mM potassium acetate at pH 7.4, 0.5 mM EDTA, and 20% glycerol. Small volumes of various concentrations of azamulin in DMSO or ethanol, tiamulin (Sigma) in DMSO, pleuromutilin (Toronto Research Chemicals) in DMSO, retapamulin (Sigma) in DMSO, and lefamulin acetate (Selleckchem.com) in DMSO were added to the sample and reference cuvettes followed by equilibration for 3 min before initiating data collection. The digital spectra were sampled at 1 nm intervals for 1 s. Excel was used to subtract the initial spectrum from subsequent spectra with volume corrections for substrate addition. Peak-to-trough differences in absorption *versus* concentration were analyzed using SlideWrite with nonlinear regression to fit the Hill equation (Equation 1), one-site binding (Equation 2), or the Morrison equation for a one-site tight binding model (Equation 3), where Δ*A*_max_ is the maximum peak-to-trough change in absorbance, L and P are the ligand and P450 concentrations, *K*_*d*_ is the dissociation constant, and the exponent *n* is the Hill coefficient (Equation 1). SlideWrite was used for nonlinear regression to fit the tight-binding equation (Equation 3) to the tiamulin data, and the one-site binding equation (Equation 2) was used for the pleuromutilin-, retapamulin-, and lefamulin-binding data.

(1) $$\Delta A=\Delta A_{max\;}\frac{L^{n}}{\left(L^{n}+{K_{d}}^{n}\right)}$$

(2) $$\Delta A=\;\Delta A_{max}\frac{L}{L+K_{d}}$$

(3) $$\text{Δ}A=\text{ Δ}A_{\text{max }}\frac{\left(P+L+K_{d}\right)-\sqrt{{\left(P+L+K_{d}\right)}^{2}-4PL)}}{2P}$$

An alternative computational approach was used for modeling the binding of azamulin. The one-site binding (Equation 2), the two-site Hill equation (Equation 1) with η = 2, the two-site sequential binding (Equation 4), and the two-site random binding (Equation 5) models were analyzed and compared using Dynafit (21), which employed numerical methods and quality comparisons to guide selection of the model. This approach includes computation of concentrations of bound and free azamulin to predict dissociation constants for equilibrium of the binary or ternary complexes and the fractional contribution of each complex to the total change in absorbance. The concentration of the P450, the concentrations of azamulin with uniform units of concentration (micromolar), and the corresponding Δ*A* values are inputs. Initial values for *K*_*d*_s and RSRs for each complex are provided. Ligand-free P450 was assigned an RSR of zero. The output includes predicted values for *K*_*d*_s and RSRs, correlations between parameters, and several statistical values for goodness of fit and variances. In addition, graphs of the fit of the data to the model and residuals are produced in several formats. When multiple models are tested in the same run, the program can output comparisons based on information theory.

(4) $$\begin{array}{l} \text{P}450\;+\text{ A <}==\text{> P}450.\text{A : K}1\text{ dissoc RSR}1\\ \text{P}450.\text{A }+\text{ A <}==\text{> A.P}450.\text{A : K}2\text{ dissoc RSR}2 \end{array}$$

(5) $$\begin{array}{l} \text{P}450\;+\text{ A <}==\text{> P}450.\text{A : K}1\text{ dissoc RSR}1\\ \text{P}450\;+\text{ A <}==\text{> A.P}450\text{ : K}2\text{ dissoc RSR}2\\ \text{P}450.\text{A }+\text{ A <}==\text{> A.P}450.\text{A : K}3\text{ dissoc RSR}3 \end{array}$$

The solutions for the two-site random binding (Equation 5) were rejected because of the wide variability observed for estimation K1 and K2 and RSR1 and RSR2. The lack of two distinct independent binding sites suggests that the sequential binding model is appropriate. The two-site sequential binding model for azamulin provided a better fit to the data than the one-site model or the two-site Hill equation based on an information theoretic analysis. The fit was also better than the algebraic fit to the Hill equation using SlideWrite.

## Data availability

Structure factors and coordinates were deposited in the Worldwide PDB with accession code 7SV2. In addition to the other data shown in the article, replicate datasets for ligand binding are available from the corresponding author: Eric F. Johnson, Department of Molecular Medicine, Scripps Research, 10550 North Torrey Pines Road, La Jolla, CA 92037, USA; johnson@scripps.edu; Tel.: 858-784-7918.

## Conflict of interest

The authors declare that they have no conflicts of interest with the contents of this article.

## Footnotes

1 Parentheses refer to PDB identification codes for a protein structure with four characters and three characters for ligands.

2 The PDB entry for the structure of azamulin (MWY) is designated (3aS,4R,5S,6R,8R,9R,9aR,10R)-6-ethyl-5-hydroxy-4,6,9,10-tetramethyl-1-oxodecahydro-3a,9-propanocyclopenta[8]annulen-8-yl [(5-amino-1H-1,2,4-triazol-3-yl)sulfanyl]acetate. The National Center for Biotechnology Information PubChem entry (compound ID number: 16072188) for azamulin is numbered differently: ((1S,2R,3S,4R,6R,7R,8R,14R)-4-ethyl-3-hydroxy-2,4,7,14-tetramethyl-9-oxotricyclo[5.4.3.0 ∼ 1,8∼]tetradec-6-yl [(5-amino-1H-1,2,4-triazol-3-yl)sulfanyl]acetate). The latter is consistent with numbering for pleuromutilin and tiamulin.
